# Applications of Extracellular Vesicles in Abdominal Aortic Aneurysm

**DOI:** 10.3389/fcvm.2022.927542

**Published:** 2022-05-31

**Authors:** Shan Lu, Ruihan Wang, Weiguo Fu, Yi Si

**Affiliations:** ^1^Department of Vascular Surgery, Zhongshan Hospital, Fudan University, Shanghai, China; ^2^Vascular Surgery Institute of Fudan University, Shanghai, China; ^3^National Clinical Research Center for Interventional Medicine, Shanghai, China

**Keywords:** aortic abdominal aneurysm, extracellular vesicle, biomarker, therapeutic, engineering

## Abstract

Abdominal aortic aneurysm (AAA) is a localized expansion of the abdominal aorta which can lead to lethal complication as the rupture of aortic wall. Currently there is still neither competent method to predict the impending rupture of aneurysm, nor effective treatment to arrest the progression of small and asymptomatic aneurysms. Accumulating evidence has confirmed the crucial role of extracellular vesicles (EVs) in the pathological course of AAA, acting as important mediators of intercellular communication. Given the advantages of intrinsic targeting properties, lower toxicity and fair stability, EVs show great potential to serve as biomarkers, therapeutic agents and drug delivery carriers. However, EV therapies still face several major challenges before they can be applied clinically, including off-target effect, low accumulation rate and rapid clearance by mononuclear phagocyte system. In this review, we first illustrate the roles of EV in the pathological process of AAA and evaluate its possible clinical applications. We also identify present challenges for EV applications, highlight different strategies of EV engineering and constructions of EV-like nanoparticles, including EV display technology and membrane hybrid technology. These leading-edge techniques have been recently employed in multiple cardiovascular diseases and their promising application in the field of AAA is discussed.

## Introduction

Abdominal aortic aneurysm (AAA) is mainly characterized with immune cell infiltration, extracellular matrix (ECM) degradation and apoptosis of vascular smooth muscle cells (VSMCs), leading to the weakening of vascular wall and dilation of abdominal aorta. The prevalence of AAA reached up to 8% among males aged over 65 years old, and the major complication of AAA is aortic rupture, of which the mortality rate exceeds 80% and causes 150,000–200,000 deaths annually across the world (1, 2). Although computed tomography angiography (CTA) and other advanced imaging techniques can provide accurate anatomic information of AAA such as its location and size, most patients of AAA are asymptomatic before the rupture occurs and the progression of AAA toward rupture is not linear but unpredictable for medical imaging techniques, so sensitive and specific biomarkers are needed to assess the condition of AAA and prevent impending rupture. According to current guidelines, large asymptomatic AAAs (>55 mm diameter in men and >50 mm diameter in women) and symptomatic AAAs are recommended for open repair surgery or endovascular aortic repair (EVAR) (3). In contrast, there are no available therapy options for small asymptomatic AAAs except for regular follower-up and monitoring of the change of AAAs. Currently no convictable evidence can support that commonly used drugs for AAA, such as β-blockers, angiotensin-converting enzyme inhibitors and antiplatelet agents, are beneficial to the limitation of AAA growth or rupture (4). In such a scenario, the need to identify alternative approaches should be prioritized.

Extracellular vesicle (EV), an important medium for intercellular communication, has drawn researcher's attention in the last few decades and been placed in the limelight in different fields of diseases. EV was first identified in differentiated reticulocytes by Pan et al. (5) in the 1980s but was only regarded as “cellular garbage bags” for expired and degenerative proteins until 2007, when Hadi Valadi et al. (6) discovered that EV contains both mRNA and microRNA (miRNA) that can be transferred to and act on other cells. Since then, a series of studies have reported that EV plays an important role in various stages of the pathogenesis of atherosclerosis, myocardial infarction (MI), ischemia-reperfusion and other cardiovascular diseases (7–9). Exosome is derived from the fusion of intracellular endosomes with the plasma membrane and represents the smallest kind of EV, which is generally 30–150 nm in diameter (10). Despite its small size, exosome is the most important subtype of EV, playing a vital role in mediating cell-to-cell communication. The current review aims to summarize the crucial role and clinical transformation value of exosome/EV in AAAs as a promising source of biomarkers, therapeutic agents and drug delivery carriers.

## The Biogenesis and Performance of EVs in AAA Development

**Figure 1:**
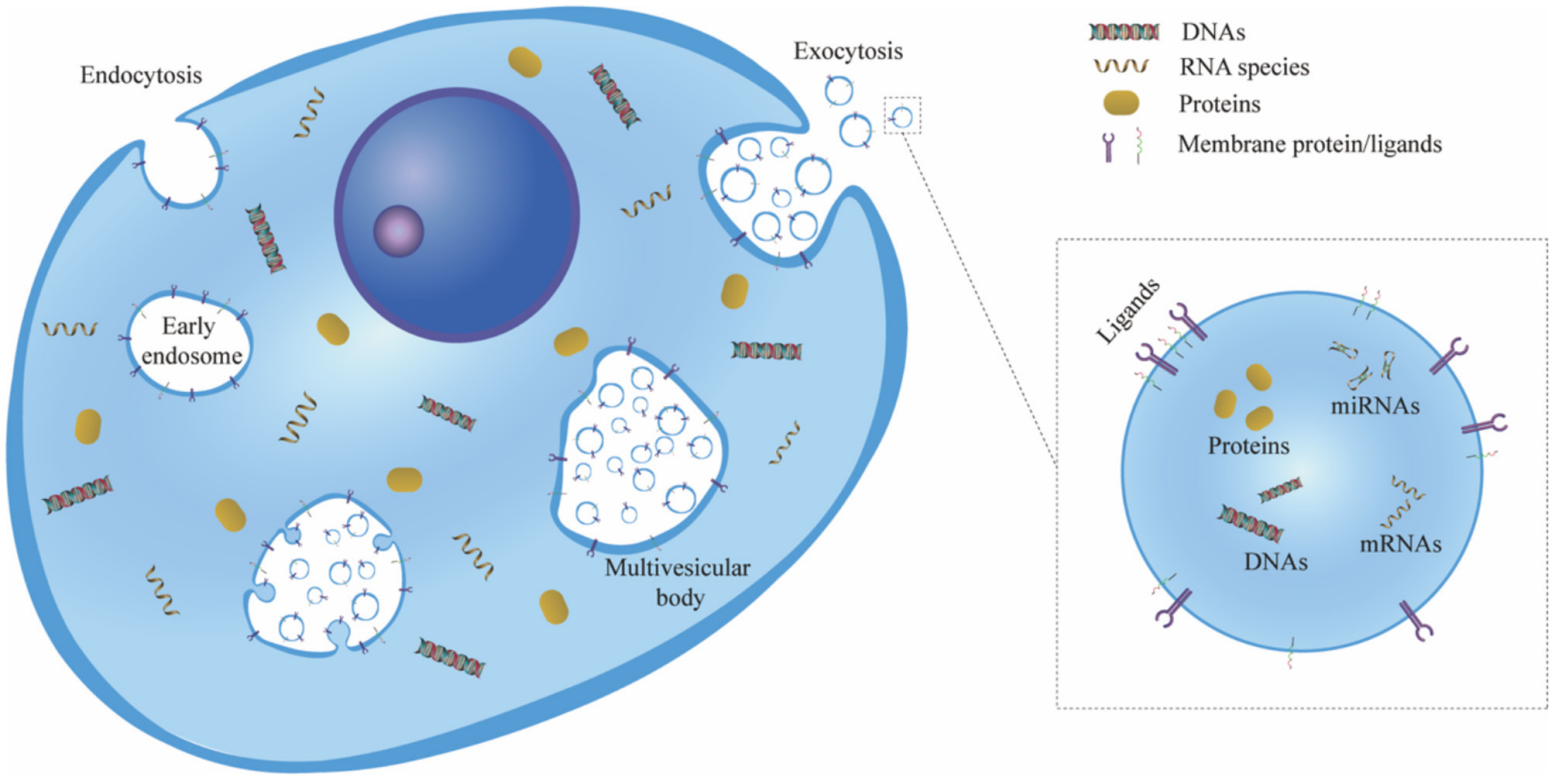
Schematic representation of exosome biogenesis. First, the cell plasma membrane buds inward to form early endosomes via endocytosis. Then, the endosome membrane invaginates and sprouts to form ILVs, and the early endosomes mature and become MVBs. Exosomes are considered ILVs that are released to the extracellular environment owing to the exocytosis of MVBs. ILV, intraluminal vesicle; MVB, multivesicular body.

EVs consist of a phospholipid bilayer envelope structure with a characteristic cup-shaped appearance, and they can be secreted by almost all types of cells and distributed throughout body fluids, including plasma, saliva, cerebrospinal fluid, lymphatic fluid and urine (11–14). The biogenesis of EVs is generally initiated in the endosome system, in which case they are referred to as exosomes. First, the plasma membrane buds inward and fuses with the primary endocytic vesicles to form early endosomes. Then, the endosome membrane invaginates and sprouts to form multivesicular bodies (MVBs) which contain multiple intraluminal vesicles (ILVs). Exosomes are considered as ILVs that are released to the extracellular environment after the fusion of MVBs with the plasma membrane (12, 15) (Figure 1).

After EVs are secreted from cells, they can stably exist in the interstitial fluid, owing to their lipid bilayer structure, which can not only protect EVs themselves from degradation but also ensure the integrity and security of the cargoes carried within. During the process of EV biogenesis, a series of molecular contents with cell biological activity are encapsulated into EVs, such as proinflammatory and anti-inflammatory cytokines, nucleic acids (DNA, RNA, mRNA, miRNA), enzymes and many other proteins (16, 17). The composition of regulatory substances carried by EVs largely depends on the cell type and state of the secretory cells (18). When cells are exposed to hypoxia, inflammation or other stressors, the components of regulatory substances will change, eventually leading to high heterogeneity in the content and types of regulatory substances in EVs. Therefore, changes in the type and quantity of regulatory substances in EVs may reflect the physiological or pathological status of parent cells (6).

EVs involve in multiple pathological processes by modulating cell-cell interaction, which significantly contributes to the progression of AAA. As mentioned above, immune cell infiltration is one of the major characteristics of AAA, and macrophages are found to account for the largest proportion of the infiltrated cells according to a recent single-cell RNA sequencing results (19). These macrophages are recruited mainly to the adventitia and media of the aortic wall and most of them will switch to M1 phenotype (20). The M1 polarization of macrophages will enhance inflammatory response and deteriorate vasculature remodeling by secreting matrix metalloproteinase (MMP), pro-inflammatory cytokines and EVs. Wang et al. (21) revealed that VSMCs incorporate these EVs and implement the messages received from macrophages. The stimulated VSMCs promote the expression of MMP-2, which would degrade surrounding ECM and further vitiate the aortic wall. However, the administration of GW4869, a widely used blocker of EV secretion, can reverse the disruptive effect of dilated aortic, which again verifies that EVs play an important part in the development of AAA. Besides, T cells are another major subset in human AAA, and a positive relationship has been observed between the T cells infiltration and AAA size (22). Dang et al. (23) found that T cell-derived EVs can promote the macrophage migration from circulatory system to aortic wall and subsequently potentiate AAA. In spite of the discovery of the roles of EVs in macrophage-VSMC and T cell-macrophage communication, the functional materials inside EVs, their relevant signal pathways and many other types of involved EVs still remain unclear and need further investigations.

## EV-Based Biomarkers and Therapies in AAA

At present, the diameter of the aorta measured by CTA is considered as the golden standard for AAA diagnosis, and regular follow-ups are required to monitor potential changes of AAA diameter. However, the progression of AAA follows a discontinuous pattern, which may accelerate unexpected between adjacent two follow-ups and cause serious adverse events. In such condition, the identification of effective biomarkers may help to predict the rupture of AAA and avoid potential death. EVs appear to be an intriguing source of biomarkers, for the changes of their circulatory numbers and contents can convey important information and reflect the pathological status of diseases. Martinez-Pinna et al. (24) performed a differential proteomic study based on human plasma-derived EVs and compared the differential expression of proteins in EVs between AAA population and normal population. The number of identified proteins in EVs is higher in AAA group than in normal group, which may indicate a hyper-secretive condition in AAA disease. And the study observed a series of proteins with enhanced expression level including ferritin, mitochondrial Hsp60, c-reactive protein and platelet factor 4, which are all closely related to AAA-relevant pathological mechanisms. Ferritin and Hsp60 involve in the oxidative stress and iron deposition in lesion area (25, 26), while c-reactive protein and platelet factor 4 are important participators in the process of inflammatory response and intraluminal thrombogenesis. Fernandez-García et al. (27) also found that ficolin-3, a crucial recognition molecule in the lectin pathway of the complement system, has an increased expression level in EVs separated from AAA patients' serum (28). Moreover, the expression level of ficolin-3 is also found upregulated in EVs isolated from the aortic wall of aneurysm and intraluminal thrombus (ILT) compared to normal aortic wall. This discovery indicates that enhanced ficolin-3 expression in serum is a result of active production and secretion in lesion tissues. Apart from proteins, miRNA is another critical cargo of EVs. It can interact with mRNAs to regulate the protein synthesis activity. Diverse miRNAs can be selectively packed into EVs and transferred from cells to cells to modulate disease-related processes. Recent research enriched EVs from serum of AAA patients and performed small RNA-sequencing to identify potential miRNA biomarkers (29). It turns out that the expressions of miR-122-5p, miR-2110 and miR-483-5p are upregulated in AAA patients' serum. However, to date no individual biomarker has been proven to be sufficient to predict such a complex disease as AAA, so the use of a combination of multiple biomarkers can be a promising approach for clinical application (30).

As in many other cardiovascular diseases, inflammatory response plays a vital role in the development of most AAAs. Chronic AAA is featured with recruitment of large amounts of immune cells, particularly macrophages and T cells, and subsequent ECM degradation and destruction of the aortic wall. The potential of stem cell therapies in AAA treatment has aroused great interest. By injecting stem cells from multiple sources (such as bone marrow, adipose and placenta) intravenously or directly to the adventitia of aortic wall, the development of AAA is evidently restricted (31–34). Further studies suggest the therapeutic effect of stem cell administration comes from the secreted EVs which convey complicated paracrine signals. Growing evidence has confirmed that EV therapy is superior to cell-based treatment in several aspects. While low retention and survival rates limit the progression of stem cell administration, the EV membrane derived from their parent cells ensures high efficiency of transmission (35). EVs can be locally transplanted in the demanded time and space with a defined and accurate dosage, whereas implanted stem cells may undergo apoptosis and subsequently have a low arrival rate to the target area (36). In addition, EVs are more stable for *in vitro* conservation and transportation as non-vital vesicles and more durable for long-term cryopreservation and freeze-thaw processes with little change in their biochemical activities (37). Most importantly, EV therapy prevails over cell-based treatment based on the principle of safety assurance. Burgeoning awareness and concern have been discussed with regard to the safety issues that arise from cell-based treatments, such as tumorigenicity, immunological rejection and occurrence of embolism (38, 39). By contrast, EVs have higher safety and better tolerability due to their low mutagenicity and low immunogenicity. Sun et al. (40) transplanted EVs derived from human umbilical cord mesenchymal stem cells (MSC) into rabbits, guinea pigs and rats, and no adverse effect were observed with respect to liver or renal function, hemolysis, vascular and muscle stimulation, systemic anaphylaxis, pyrogen or hematology indexes. Clinical trials of EV administration in cancer patients also revealed positive therapeutic outcomes without adverse effects (41, 42).

The therapeutic potential of stem cell-derived EVs has been extensively explored in multiple cardiovascular diseases, especially in myocardial infarction and atherosclerosis (43–47). However, there is currently only a few researches about the application of EV in AAA treatment. Sajeesh et al. (48) investigated the effects of EVs derived from bone marrow-derived MSC (BM-MSC) in the context of AAA rat model. It appears that EVs can tip the balance of the aortic lesion from a proteolytic milieu to an anti-proteolytic one, mainly by suppressing the expression of elastolytic MMP2 and enhancing the expression of natural tissue inhibitor of MMP2 (TIMP-2). Intriguingly, it's reported that MMP2 is primarily overexpressed in small AAAs, whereas large, rupture-prone AAAs mainly exhibit up-regulation of MMP-9 (49, 50). This indicates potential therapeutic value of BM-MSC-derived EVs in the early treatment of small AAAs. Intravenous injection or local administration of therapeutic EVs may help to slow down the course of AAA. As mentioned above, miRNA is a principal cargo of EVs and is closely involved in AAA development. EVs from different cells have diverse miRNA expression profiles and multiple miRNAs have been reported to exert different effects on disease progression. Spinosa et al. (51) defined the critical role of MSC-derived EVs in attenuating the aortic dilation and relieving inflammatory response via miR-147. And the transfection of miR-147 inhibitor in MSC abrogates such therapeutic effect, which in turn confirms the critical role of miR-147. Contrariwise, some miRNAs contribute to the progression of AAA formation. For example, miR-106a is found to down-regulate the expression of TIMP-2 and accelerate VSMC cell apoptosis and ECM degradation (52). Similarly, overexpression of miR-29b appears to augment AAA expansion in a mice model, while the administration of anti-miR-29b triggers a fibrotic response and retards AAA growth (53). And in human AAA tissue samples, a reduction of miR-29b expression is also observed, which may suggest the down regulation of miR-29b is a physiological protective response of the aortic wall to expansion. Local delivery of anti-miRNA drugs via expandable balloons and drug-eluting stents can be innovative avenues for small AAA treatment (54).

Altogether, as important mediators of intercellular communication, EVs can incorporate bioactive molecules and are endowed with intrinsic targeting properties and low immunogenicity because of their inherited membrane from parent cells (55). These characteristics make EVs a more promising drug delivery system than traditional synthetic delivery system (56). However, there are several disadvantages hindering further utilization of EVs in a therapeutic context. Innate EVs lack of specific molecules to exclusively bind with target tissue or cells and their low accumulation rate also adds to the off-target effect (57). Another major challenge is the noteworthy loss rate of circulating EVs due to mononuclear phagocytosis, which leads to rapid clearance and maldistribution of EVs (58). Therefore, different strategies for EV engineering are developed and constructions of bioinspired EV-like nanoparticles are elaborately designed to obtain higher delivery efficiency and better therapeutic effect.

## Recent Advancement in the Engineering of Therapeutic EVs

This section will highlight the current promising strategies of EV engineering technologies. Because only very few researches have focused on EV engineering in the field of AAA treatment, we will first broaden our outlook and introduce engineering strategies applied in cardiovascular diseases, and then proposed feasible utilizations in the area of AAA.

### EV Display Technology

Although the many advantages of EV bring it further attention as a drug delivery vehicle, an unsatisfactory targeting rate still hinders the clinical use of EV therapies. Natural unmodified EVs are enriched in and cleared by the liver, spleen, kidney and other organs after systematic administration, making it difficult to achieve effective therapeutic concentrations in target organs (59). Selecting an efficient engineering strategy is a necessary step to further improve EV-based therapies. EV display technology, which allows re-engineering of the membrane protein composition, has been extensively studied in the last decade (60).

A popular application of EV display technology is to add targeting ligands via transfection of parent cells with fusion genes of targeting peptides and EV membrane proteins. The overexpression of the fusion protein on the surface of EV membrane can steer the engineered EVs directly toward target area. Lysosomal-associated membrane protein 2 (Lamp2b) is expressed abundantly on the surface of EV, which is extensively chosen as a component of the designed fusion protein (61). Wang et al. (62) engineered Lamp2b fused with ischemic myocardium-targeting peptide CSTSMLKAC (IMTP) to treat myocardial infarction. They found the bioengineered EVs can specifically target ischemic myocardium and exert therapeutic effects on acute myocardial infarction. Similarly, cardiac-targeting peptide (CTP)-Lamp2b is generated and expressed on the EV membrane and it can enhance EV delivery to heart cells and tissue without toxicity (63). In the context of AAA, the features of aneurysm lesions can be utilized as potential targets, involving inflammatory cell infiltration, MMP overexpression, ECM degradation and VSMC apoptosis. Elastin constitutes the tunica media of aortic wall, which is always found degraded in the pathological progression of AAA. Elastin fiber is mainly composed of two components, which are a core of amorphous cross-linked elastin protein and peripheral fibrils (64). Before the degradation of elastin protein, MMPs will first break down the peripheral glycoproteins and thus expose the hydrophobic core of elastin (65). Sinha et al. (66) took advantage of this pathological feature and developed an elastin antibody tethered nanoparticle (EL-NP) to target degraded elastic lamina. They observed in rat AAA model that the EL-NPs can specifically target AAA after systematic administration, and accumulate in the degraded elastic lamina rather than healthy aorta. Inspired by this, it is plausible to fuse the genes of elastin antibody with Lamp2b, and the overexpressed fusion protein can guide the therapeutic EVs toward target tissue and cells in the dilated aorta. Besides elastin, other characteristic molecules and cells such as collagen, MMP, VSMC and macrophages can also be considered as potential delivery targets for EV engineering (67, 68).

Targeting molecules can also be added to the surface of the EV membrane via a direct chemical engineering approach. Membrane coating technology can provide an excellent platform for the insertion of targeting peptides into EV membrane (69). Polymeric materials, such as polylactic-co-glycolic acid (PLGA) and polyethylene glycol (PEG), are first cocultured with targeting peptides. Then parent cells or EVs are incubated with the polymeric materials-peptide mixture. Polymeric materials can self-assemble to form the membrane-coated structure with anchored targeting peptides, which provide EVs with extended circulation time and promoted targeting ability (70). Wang et al. (71) attached the Arg-Gly-Asp (RGD) peptide to the surface of EVs via linkage to PEG-lipid, which self-assembles into the EV membrane. Since the RGD peptide can specifically bind to integrin α_V_β_3_ expressed on the surface of angiogenic blood vessels, RGD can guide bioengineered EVs to blood vessels and promote therapeutic angiogenesis. Likewise, Vandergriff et al. (72) conjugated cardiac homing peptide (CHP) with dioleoylphpatidylethanolamine N-hydroxysuccinimide (DOPE-NHS) and incubated the DOPE-CHP complex with isolated EVs. The lipophilic tails of DOPE-CHP can spontaneously insert into the EV membrane, thus coating EVs with the CHP peptide. The results demonstrated that the engineered EVs exhibit improved viability and elevated targeting ability, which lead to reductions in fibrosis and scar size at the infarcted site and promotion of cellular proliferation and angiogenesis. Similar strategy can be applied to the implantation of elastin antibody or other targeting peptides on EV membrane in AAA treatment. This chemical-based engineering approach elicits a similar effect on the promotion of delivery efficiency compared to the parnet-cell-based methods (73). However, the risk of a possible immune response to synthetic polymers concerns researchers. Despite the surface functionalization provided by the inserted peptides, it's difficult to accurately simulate the complex interfaces of the natural cell membrane merely by adding certain targeting proteins or peptides. More recently, researchers have shifted their interests to utilizing natural cell membrane components as EV coating materials.

**Figure 2:**
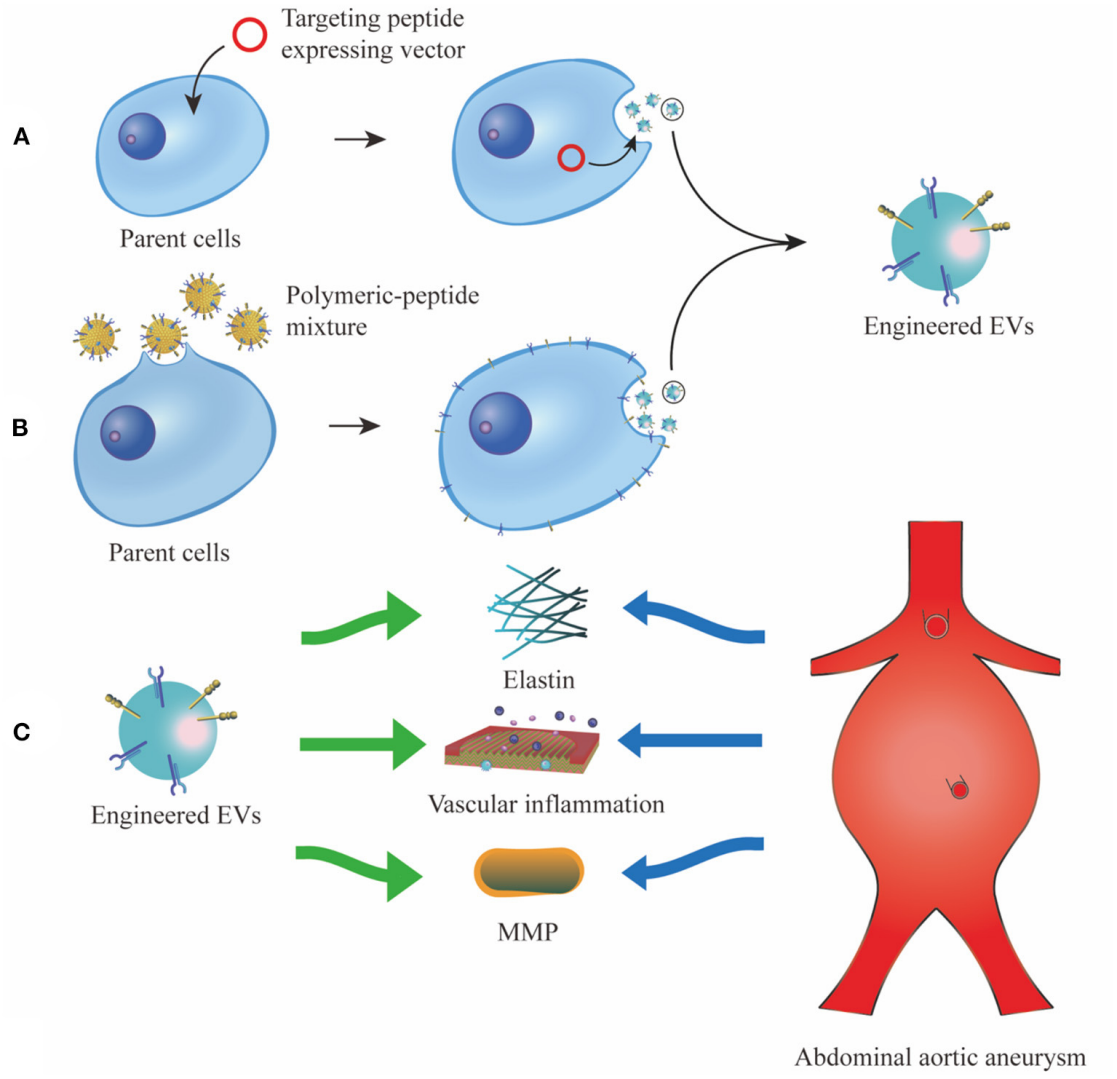
Different strategies of EV display technology. **(A)**, vectors which convey fusion genes of targeting peptides and EV membrane proteins are transfected into the parent cells. The targeting peptides are overexpressed and enriched on the surface of EV membrane. **(B)**, targeting peptides are first cocultured with polymeric material, then the polymeric-peptide mixture self-assembles to the membrane of parent cells and their derived EVs. **(C)**, the engineered EVs can specifically target characteristic tissue and cells in AAA, such as elastin, vascular inflammation and MMP. EV, extracellular vesicle; MMP, matrix metalloproteinase.

In addition to targeting proteins or peptides, other specific compounds, such as magnetic nanoparticles and nucleic acid aptamers, can also be applied to direct exosomes toward target cells and tissues (74–76). Taken together, above-mentioned strategies enhance the targeting abilities of EVs by displaying or modifying specific molecules on the surface of EV membrane (Figure 2). However, EV display technology is not without issues. For example, targeting peptides displayed on the surface of EV membrane are sometimes degraded by proteases in the cells or body fluids, resulting in a loss of their targeting ability. Moreover, if the relative molecular weight of targeting peptide is too high, the expression or correct folding of the fusion protein could be disrupted, thereby restricting its effect on EV functions. Therefore, it is of great significance to enhance the durability of targeting peptides to biodegradation and reduce their relative molecular weight to further develop EV display technology.

### Membrane Hybrid Technology

As we discussed above, most naturally secreted EVs lack sufficient targeting capability. To achieve a desired therapeutic effect, a higher dosage of EVs is used, but this creates another dilemma in which large amounts of EVs injected in the circulatory system will accumulate in the liver and kidney and cause a series of toxic responses (77). Although adding targeting proteins or peptides on the surface of EV membrane can largely enhance homing efficiency, this approach might be suboptimal since these modifications are often highly labor-intensive and time-consuming processes. Besides, the addition of several functional membrane proteins can hardly simulate the complex interface of the natural cell membrane and the protein-protein interaction network. Cell membrane-camouflaged nanoparticles, which combine the versatile functionalities of different types of cellular membranes with therapeutic nanomaterials, have recently gained increasing attention (78). Membranes from various kinds of cells, such as erythrocytes, leukocytes, platelets, stem cells and even cancer cells, are exploited as carriers to transport drugs to treat a multitude of diseases (70, 74, 78–81). Cell membrane-camouflaged technology has emerged as an interesting biomimetic strategy to imbue nanomaterial with the inherent functions and properties of natural cells for various biomedical applications. Inspired by the cell membrane-camouflaged strategy, biomimetic engineering has been shown to hybridize EV membrane with different cell membranes, which has recently emerged as a novel research avenue to promote the efficiency of EV delivery in various cardiovascular diseases.

Leukocyte infiltration is often a hallmark of inflammatory response in cardiovascular diseases, and monocytes from the circulatory system are the main cell type that infiltrate lesion area and orchestrate tissue remodeling (82). After they are recruited to the injury site, monocytes then differentiate into macrophages and localize to the lesion, playing an important role in ECM remodeling and removal of dead cell debris (20, 83). Multiple adhesive molecules collectively regulate the migration of monocytes, including macrophage receptor 1 (Mac1; also known as integrin αMβ2), P-selectin glycoprotein ligand 1 (PSGL1), very late antigen 4 (VLA4; also known as integrin α4β1) and C-C motif chemokine receptor 2 (CCR2) (84, 85). To utilize their chemotaxis wandering ability, monocytes are processed by cell lysis, differential centrifugation and homogenization to form monocyte membrane vesicles, which are later hybridized with prepared EVs via an incubation-extrusion process. Besides *in vivo* homing ability, monocyte membrane also contains an important signaling protein, CD47, which can act against opsonization and reticuloendothelial system (RES) clearance, thus providing membrane-hybrid EVs with immune evasion ability (86). Zhang et al. (87) constructed monocyte-membrane-hybrid MSC-EVs (Mon-EVs) in a mouse myocardial ischemia-reperfusion injury model. Mon-EVs had a longer circulation time and higher accumulation rate at the lesion than unmodified EVs did, and Mon-EVs treatment exhibited a more favorable effects on cardiac function and remodeling, neovascularization and endothelial maturation. In the context of human AAA, proinflammatory macrophages are mainly found in the adventitial layer of aortic aneurysm (88). The recruitment of circulatory macrophages to the lesion area largely relies on selectins and multiple chemokine-receptor pathways, especially CCL2/CCR2 axis (89–91). Deficiency of any signaling receptor or ligand has been proved to decrease macrophage accumulation in the aortic wall and reduce aneurysm formation (92–94). The fusion of MSC-derived EVs with monocyte membranes can theoretically endow the hybridized products with an enhanced active targeting efficiency toward the adventitial lesion of AAA, and subsequently achieve a better therapeutic effect.

Platelets are unique anucleate cell fragments which involve in many pathophysiological processes, including atherosclerosis, tumor development and inflammation (95). In circulation, platelets can rapidly adhere to location of vascular lesion and aggregate to form hemostatic plugs. The lipid bilayer of platelet membrane is festooned with transmembrane proteins and other glycoprotein integrins, including membrane binding molecules such as GPIIb/IIIa and CD62P, and immunomodulatory proteins such as CD47 and CD55 (96, 97). The GPIIb/IIIa complex, also known as integrin αIIbβ3, is one of the most abundant receptors expressed on the platelet membrane and plays a pivotal role in mediating platelet aggregation (98). Given the advantages of platelet membrane, Hu et al. (99) constructed platelet-membrane-hybrid exosomes (P-XOs) in the treatment of MI mouse model. Compared to non-modified EVs, P-XOs showed remarkably enhanced cardiac targeting ability because of transmembrane proteins GPIV and GPIX, and integrin-associated tetraspanins CD9 and CD81 on the platelet membrane. Immunomodulatory proteins such as CD47 and CD59 help P-XOs bypass macrophagic clearance, leading to extended circulation time. Consequently, P-XOs exhibited improved therapeutic capacity to promote angiogenesis, inhibit oxidant injury in cardiomyocytes and ameliorate cardiac function. In the context of AAA, ILT is present in about 75% of all AAAs, which may have both accelerative effect and protective effect on AAA growth and its potential rupture (100). On one hand, ILT is found to harbor large quantities of inflammatory cells and proteases, which degrade AAA matrix and further weaken the aortic wall (101, 102). On the other hand, ILT provides physical cushioning for AAA wall from high hemodynamic stresses, thus protecting patients from abrupt AAA rupture (103, 104). Based on these factors, it's important to steadily dissolve ILT to reduce its proteolytic effect while stabilizing its mechanical shielding structure to protect AAA wall. Sivaraman et al. (105) encapsulated tissue plasminogen activator inside PLGA-nanoparticles and found the slow release of tissue plasminogen activator can gradually lyse ILT without damaging its general structure. Also, the porous structure of ILT facilitates the penetration of therapeutic drugs from circulation toward AAA wall. Further, Pawlowski et al. (106) developed platelet microparticle-inspired nanovesicles, which mimic the membrane interface structure of platelet-derived EVs. These engineered nanovesicles turned to specifically anchor onto thrombus via active platelet integrin GPIIb/IIIa and P-selectin, and then accurately release their thrombolytic payload. Since the platelet membrane can supply targeting ability toward thrombus, it's a plausible approach to load MSC-derived EVs with thrombolytic drugs and hybridize them with platelet membranes, in order to achieve a therapeutic effect at both the ILT and AAA wall.

**Figure 3:**
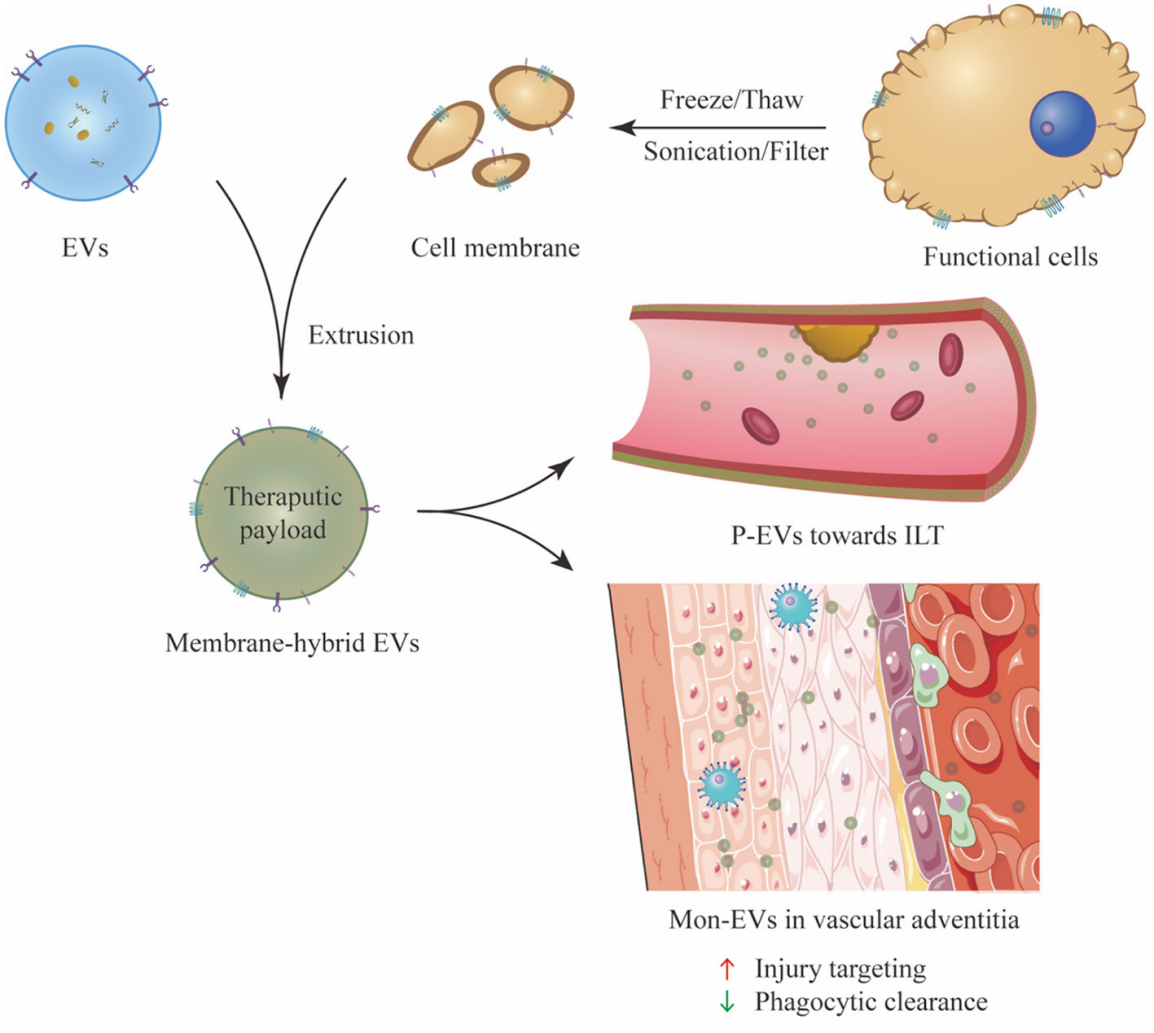
Preparation and experimental application of membrane hybrid technology. P-EVs can actively target the ILT which adheres to the AAA wall, while Mon-EVs mainly accumulate in the adventitia of aortic wall and exert therapeutic effect. The membrane-hybridized EVs exhibit enhanced injury targeting ability and immune evasion ability. P-EV, platelet-membrane-hybrid EV; ILT, intraluminal thrombus; Mon-EVs, monocyte-membrane-hybrid EV.

Membrane hybrid technology has emerged as a novel and versatile strategy to endow EVs with desirable functions and a complex surface interface similar to cell membranes, thereby providing improved drug delivery efficiency; this would otherwise be unachievable via peptide conjugation technology. This method is generalizable to all kinds of EVs with simple procedures of membrane fusion, and it may advance engineered EVs as a promising and preferable tool in the treatment of AAA (Figure 3).

## Conclusions

The onset mechanism and different treatments for AAA have gradually been studied in depth, yet to date the mainstream therapies only involve surgical or endovascular repair applied for large or symptomatic AAAs. Effective treatments for small AAAs are absent, and a sensitive monitoring method is needed to predict potential AAA rupture and disease progression. As an important mediator of intercellular communication, EVs offer exciting promise for monitoring and treatment in AAA and possess various advantages, such as membrane stability, biocompatibility and intrinsic targeting properties. The bioactive molecules inside EVs, including cytokines, enzymes and nucleic acids, especially miRNAs, play a vital role in mediating immune cell infiltration, MMP expression, EMC degradation and VSMC apoptosis. Different drugs can be loaded into EVs or EV-like nanoparticles and delivered toward the lesion to slow, arrest or even reverse the AAA growth (107–109). However, natural EVs lack sufficient targeting ability to specifically bind to the site of injury, and they are easily eliminated by the mononuclear phagocyte system. To overcome these limitations, scientists have developed different strategies, including EV display technology and membrane hybrid technology, to promote targeting ability and immune evasion ability of engineered EVs. But still, the research of EV applications for AAA is in its infancy. Current knowledge regarding the spatial and temporal release of EVs from various cells in the pathogenesis of AAA still largely remains inadequate. Different experiments are required to specify the accurate dosage and frequency of EV administration. And important issues about *in vivo* pharmacokinetic properties, mode of administration and medication safety await comprehensive assessments. To answer these questions, extensive EV research of AAA diagnosis and treatment is essential for the future translation from bench to bedside.

## Author Contributions

SL and RW conducted the literature review, drafted the manuscript, and prepared the figures. WF and YS edited and revised the manuscript. All authors have substantially contributed to the article and approved the submitted version.

## Funding

This work was supported by the National Natural Science Foundation of China (Grant 82070497 to YS).

## Conflict of Interest

The authors declare that the research was conducted in the absence of any commercial or financial relationships that could be construed as a potential conflict of interest.

## Publisher's Note

All claims expressed in this article are solely those of the authors and do not necessarily represent those of their affiliated organizations, or those of the publisher, the editors and the reviewers. Any product that may be evaluated in this article, or claim that may be made by its manufacturer, is not guaranteed or endorsed by the publisher.
